# Oxidative and pre-inflammatory stress in wedge resection of pulmonary parenchyma using the radiofrequency ablation technique in a swine model

**DOI:** 10.1186/1749-8090-7-7

**Published:** 2012-01-19

**Authors:** Theodoros Karaiskos, Periklis Tomos, Irene Asouhidou, Nikolaos Nikiteas, Theodoros Kontakiotis, Apostolos Papalois

**Affiliations:** 1 Department of Cardiothoracic Surgery "G. Papanikolaou" General Hospital, Thessaloniki, Greece; 2 2nd Department of Educational Surgery, National and Kapodestrian University of Athens, Medical School; 3 2nd Department of Anesthesiology, "G. Papanikolaou" General Hospital, Thessaloniki, Greece; 4 Pulmonary Department, "G. Papanikolaou" General Hospital, Aristotle University of Thessaloniki; 5 Experimental Research Centre, ELPEN Pharmaceuticals, Athens, Greece

**Keywords:** radiofrequency ablation, lung, MDA, TNF-a

## Abstract

**Background:**

Radiofrequency ablation (RFA) is a thermal energy delivery system used for coagulative cellular destruction of small tumors through percutaneous or intraoperative application of its needle electrode to the target area, and for assisting partial resection of liver and kidney. We tried to evaluate the regional oxidative and pre-inflammatory stress of RFA-assisted wedge lung resection, by measuring the MDA and tumor Necrosis Factor Alpha (TNF-α) concentration in the resected lung tissue of a swine model.

**Method:**

Fourteen white male swines, divided in two groups, the RFA-group and the control group (C-group) underwent a small left thoracotomy and wedge lung resection of the lingula. The wedge resection in the RFA-group was performed using the RFA technique whereas in C-group the simple "cut and sew" method was performed. We measured the malondialdehyde (MDA) and TNF-α concentration in the resected lung tissue of both groups.

**Results:**

In C-group the MDA mean deviation rate was 113 ± 42.6 whereas in RFA-group the MDA mean deviation rate was significantly higher 353 ± 184 (p = 0.006). A statistically significant increase in TNF-α levels was also observed in the RFA-group (5.25 ± 1.36) compared to C-group (mean ± SD = 8.48 ± 2.82) (p = 0.006).

**Conclusion:**

Our data indicate that RFA-assisted wedge lung resection in a swine model increases regional MDA and TNF-a factors affecting by this oxidative and pre-inflammatory stress of the procedure. Although RFA-assisted liver resection can be well tolerated in humans, the possible use of this method to the lung has to be further investigated in terms of regional and systemic reactions and the feasibility of performing larger lung resections.

## Introduction

Radiofrequency ablation (RFA) is performed by the delivery of electrical energy through a needle electrode to the area of interest, using high frequency alternating electrical current in order to cause a heat-based tissue damage. After hitting the target tissue, the current returns back to the generator through grounding skin pads. As the electrical energy increases the tissue temperature to 60-100°C there is denaturation of cellular proteins, melting of lipids, and instantaneous coagulative necrosis, resulting in irreversible cell damage.and destruction of tissue. Tissue destruction is enhanced by the creation of a secondary, wedge-shaped zone of damage due to vascular thrombosis. The RFA-created lesion heals with fibrosis and scarring [1].

This technique has been widely used for the in situ destruction of small, usually metastatic tumors, and in high risk patients [2-4]. Furthermore RFA has also been used in general surgery for the successfully completion of partial liver and renal resection due to its ability to create an extensive bloodless plane of coagulative necrosis [5-8].

In the lung, RFA is mainly used or the percutaneous treatment of small tumors, mostly under CT-guidance. To the best of our knowledge, there are still scarce data on the use of RFA in lung resection, one of these being a swine model study of our team with promising results [9]. However the local and systemic results of RFA lung resection need further investigation as lung tissue damage and coagulative necrosis created as a result of RFA thawing process can probably act as a source of toxic substances that can further induce a systemic inflammatory reaction. This has been shown in animal studies whereas RFA-assisted resection of more than 40% of the liver caused significant systemic inflammatory responses and led to poor survival [10].

In order to further evaluate the efficacy and safety of the RFA technique in performing a wedge lung resection we tried to compare the severity of primary local oxidative and pre-inflammatory stress between the RFA technique described in our previous study with the standard open thoracotomy wedge resection technique [9]. Malondialdehyde (MDA) is an end product of cellular injury and free radical formation and and an indicator of oxidative stress in cells, tissues and lung injury [11]. Tumor Necrosis Factor Alpha (TNF-α), also called cachectin, is a pre-inflammatory cytokine mainly produced by activated macrophages. Moreover several studies conducted upon cell lines, animal models and human beings suggest that TNF-α plays a primary role in the inflammatory process [12-14]. TNF-α has been also regarded as one of the major mediators of systemic progression and tissue damage in severe disease, and there is good evidence that TNF-α helps to propagate the extension of a local or systemic inflammatory process [15,16].

This study was designed to evaluate primary lung tissue concentration of MDA and TNF-α before and shortly after the wedge resection in both techniques. For our animal study we used a swine model since the swine lung is large enough to more accurately simulate the human lung.

## Materials and methods

Approval from the Ethics Committee of Animal Care of East Attica County was obtained before commencement of this study. Fourteen white male pigs with an average weight of 26 kg were divided in two groups, the RFA-group, and the control (C) group.

### Anesthesia protocol

A single dose of prophylactic cefamandole was given preoperatively. Induction to anesthesia was managed by administration of pentothal 8 mg/kg, pancuronium 0.15 mg/kg and fentanyl 17.5 μg/kg. Under general anesthesia the animals were placed in the supine position, intubated with a double-lumen endotracheal tube, and mechanically ventilated. Two large-bore vein catheters were placed for intravenous fluid and drug administration. Maintenance to anesthesia was managed by administering of pentothal at 3.5 mg/kg/h, pancuronium at 0.07 mg/kg every 20 minutes and fentanyl at 15 μg/kg/hr iv. The animals were placed on the right lateral decubitus position. Their vital signs were closely monitored during the operative procedure.

### RFA Device

For the RFA group a Radionics Cooltip Radio Frequency System (Radionics Inc., Burlington, MA) consisting of a radiofrequency generator, a peristaltic perfusion pump, grounding pads, and the appropriate electrodes was used. The electrodes were single-shaft, 15 cm long, with a 2 cm exposure tip. The mode was set to "manual" and the timer was off.

### Surgical Technique

For the RFA-group, after collapsing the left lung, a mini lateral thoracotomy was performed. After entering the thoracic cavity a small rib retractor was placed, and the lingula of the lung was retracted with a non-crashing clamp. The RFA needle electrode was inserted in the pulmonary parenchyma and the radiofrequency generator was activated by gradually increasing the output to maximum. At the end of the session a spherical zone of coagulative necrosis with a radius of 2 cm was achieved. A series of such overlapping spheres of thermal coagulation were created along the intended line of wedge resection, one next to the other, for completing the plane of resection. Following this, a lung tissue of 4 cm in diameter was divided along the ablated zone by using surgical scissors. After removal of the lung specimen, a 1 cm coagulated zone was left behind along the resection margin to ensure the sealing of blood vessels and terminal bronchial structures.

In cases of minor bleeding at the site of lung resection or air leak after lung expansion, the lung was re-excluded from ventilation, the cut surfaces were approached and additional RFA was applied with the tissue compressed together to enhance the ablation effect. Finally the lung was tested under water for air leakage with application of 20 cm H_2_O intrabronchial pressure. Covering it with parietal pleura or any other aerostatic or hemostatic technique did not reinforce the cut surface of the divided lung. With regard to the C-group, following thoracotomy the lung parenchyma of the lingula was grasped with a lung retraction clamp and pulled away. A concave, oblique, non-crashing clamp was placed proximally to the pulmonary lateral edge thus creating a 4 cm in diameter lung tissue specimen. The lung tissue peripheral to the clamp was cut with scissors and the pulmonary parenchyma left was repaired with a double row suture using prolene 4-0.

In both groups, before thoracotomy closure a small caliber chest tube was placed two interpleural spaces below the thoracotomy incision and put under gentle suction until completion of chest closure. After skin closure the chest tube was removed and a chest x-ray was done 4 hours after, for pneumothorax exclusion. All the animals were successfully extubated and after having a chest x-ray, they were transferred to their facilities.

### Specimen collection

Two tissue specimens were collected from the resected pulmonary parenchyma in both groups. One specimen was collected from the pulmonary healthy tissue before the procedure and the second one near the radiofrequency ablation line immediately after resection. The specimens were sent for TNF and MDA analysis. Samples were frozen at -70°C to prevent further loss of MDA and new sample oxidation. They were also protected from light to prevent photoxidation.

## Method of tissue analysis

### Tissue homogenization

Tissues were rinsed with ice-cold isotonic saline before homogenization. Tissue homogenization was carried out using Tris buffer 20 mM pH 7,4 and an ULTRA-TURRAX (IKA-Labortecnik) blender. 1 ml buffer was used for 0.1 gr of tissue. 10 ul of 500 mM BHT was added to 1 ml of tissue homogenate to prevent sample oxidation. The homogenate was centrifuged at 3000 × g at 4°C for 10 minutes. 0,2 ml of the supernatant was tested.

The MDA measurement was based on the reaction of a chromogenic reagent, N-methyl-2-phenylindole (MPI), with MDA at 45°C. The principle of method is that one molecule of MDA reacts with two molecules of MPI to yield a stable chromophore with maximum absorbance at 586 nm. The reagents and procedure was according to the kit BIOXYTECH LPO-586, of OXIS International, Inc. The results were expressed in μM.

The reagents and procedure, for the TNF-α measurement was according to a specific solid phase sandwich Enzyme Linked-Immuno-Sorbent Assay (ELISA) kit for swine (Sw) TNF-α. A monoclonal antibody specific for Sw TNF-α has been coated onto the wells of the microtiter strips provided. Samples, including standards of known Sw TNF-α content, control specimens, and unknowns, are pipetted into these wells. During the first incubation, the Sw TNF-α antigen binds to the immobilized (capture) antibody on one site. After washing, a biotinylated monoclonal antibody specific for Sw TNF-α is added. During the second incubation, this antibody binds to the immobilized Sw TNF-α captured during the first incubation. After removal of excess second antibody, Streptavidin-Peroxidase (enzyme) is added. This binds to the biotinylated antibody to complete the four-member sandwich. After a third incubation and washing to remove all the unbound enzyme, a substrate solution is added, which is acted upon by the bound enzyme to produce color. The reagents and procedure, for the TNF-α measurement was according to the ELISA kit KSC-3011 of INVITROGEN Corporation (CA, USA). The results were expressed in pg/ml tissue homogenate.

### Statistical Analysis

Data were expressed as mean ± SD. Differences in categorical data were evaluated using the paired student t-test. A beta error level of 20% or statistical power of 80% and a-level of 0.05 was used to calculate the sample size of this study. A value of P < 0.05 was considered to represent statistical significance. Based upon our preliminary data, a priori power analysis indicated that 5 subjects in each group would be a sufficiently large sample size to be adequate to detect a 30% alteration in MDA values, with a type-I error of 0.05 and a power of approximately 80%. Also, a priori power analysis indicated that 5 subjects in each group would be a sufficiently large sample size to be adequate to detect about 20% alteration in TNF values, with a type-I error of 0.05 and a power of approximately 80%.

## Results

None of the animals enrolled in this study was excluded from the results as there were no early postoperative deaths.

**Figure 1:**
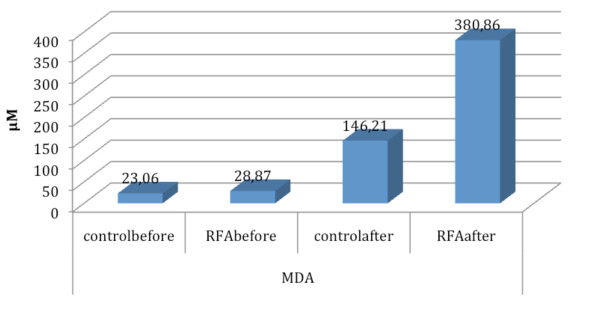
**Schematic graph of MDA mean values alterations in control and RFA group**.

**Figure 2:**
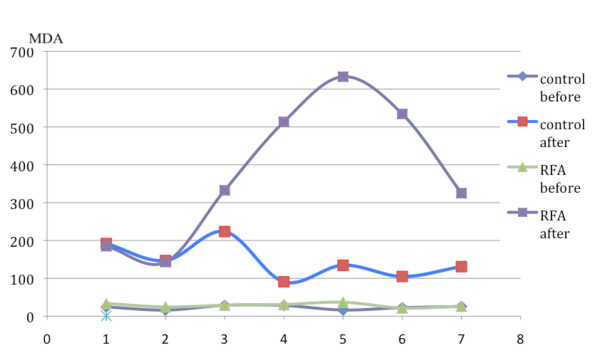
**Schematic graph of MDA mean deviation rate changes in both groups**.

MDA levels were not statistically different in two groups before wedge resection of the lung (mean ± SD C-group: 23 ± 5.16, mean ± SD RFA group: 28.9 ± 5.28, p = 0.059). Table 1 summarizes the value of MDA before and immediately after the wedge resection in control and RFA group. In C-group the mean value of MDA after resection was 146 ± 46.9 whereas in the RFA-group was 381 ± 185 (p = 0.007) (Figure 1). Also, the mean MDA deviation rate in C-group was 113 ± 42.6 whereas in the RFA group was significantly higher 353 ± 184 (p = 0.006) (Figure 2).

**Table 1:** Values of MDA in μM before and after the wedge resection of lung parenchyma in control and RFA group.

MDA	Control group			RFA group		
**No**	**Before**	**After**	**Dif**	**Before**	**After**	**Dif**

1	24.36	191.9	95.54	33.4	184.92	151.52

2	16.06	147.04	130.98	24.40	142.90	118.50

3	28.60	223.43	194.83	28.82	332.56	304.74

4	28.16	90.86	62.70	30.80	513.43	482.63

5	16.28	134.43	118.15	36.80	632.78	598.98

6	22.44	104.90	82.46	21.56	534.35	512.79

7	25.52	130.92	105.4	26.30	325.05	298.75

Mean ± SD	23.1 ± 5.16	146.1 ± 46.9		28.9 ± 5.28	381 ± 185	

p	0.007					

**Figure 3:**
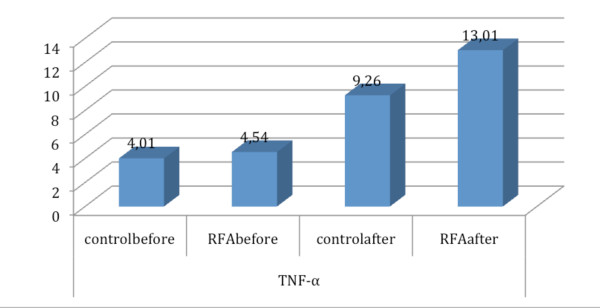
**Schematic graph of TNF mean values alterations in control and RFA group**.

**Figure 4:**
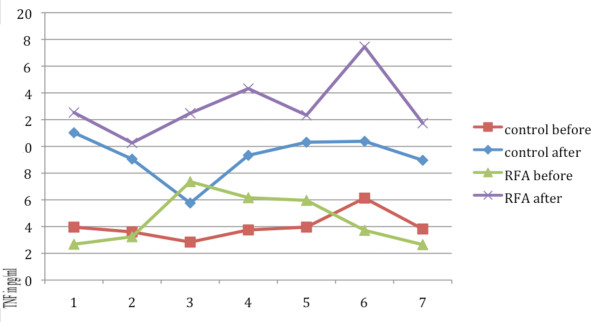
**Schematic graph of TNF mean deviation rate changes in both groups**.

TNF-α levels before and immediately after the wedge resection in the two examined animal groups are shown in Table 2. Statistically significant differences in TNF-α levels, were not observed between the two investigated groups before the wedge resection (mean ± SD C-group: 4.01 ± 1.01, mean ± SD RFA-group: 4.54 ± 1.91, p = 0.529). However, a statistically significant increase in TNF-α levels was observed in the RFA-group (mean ± SD: 13 ± 2.3) after the wedge resection compared to C-group (mean ± SD = 9.26 ± 1.72 (p = 0.005) (Figure 3). Also, the TNF-a mean deviation rate in C-group was 5.25 ± 1.36 when in the RFA-group it was significantly higher 8.48 ± 2.82 (p = 0.018), (Figure 4).

**Table 2:** Mean TNF-α levels (pg/ml) in both investigated groups before and after resection

TNF-α	Control group			RFA group		
**No**	**Before**	**After**	**Dif**	**Before**	**After**	**Dif**

1	3.96	11.02	7.06	2.68	12.53	9.85

2	3.60	9.05	5.45	3.24	10.26	7.02

3	2.84	5.76	2.92	7.36	12.48	5.12

4	3.75	9.33	5.58	6.16	14.32	8.16

5	3.96	10.31	6.35	5.96	12.33	6.37

6	6.13	10.38	4.25	3.72	17.45	13.73

7	3.82	8.96	5.14	2.65	11.73	9.08

Mean ± SD	4.01 ± 1.01	9.26 ± 1.72		4.54 ± 1.91	13 ± 2.3	

p	0.005					

## Discussion

In this experimental study we evaluated the severity of primary regional oxidative and pre-inflammatory stress of the pulmonary parenchyma at the site of wedge resection performed using RFA. As multiple applications of the RFA catheter are needed for creation of the plane of resection we hypothesized that there is enormous cell destruction that promotes oxidative processes and that the released toxic substances from the charring process can further induce a cascade of local inflammatory reaction with systemic consequences. To evaluate this oxidative and regional pre-inflammatory process we studied the tissue concentration of MDA and TNF-α before and shortly after the resection between the two animal groups.

In our study we observed a statistically significant increase of the regional oxidative stress as it is estimated by the sharp increase of MDA levels only in the RFA group. MDA is an end product of lipid peroxydation and results from degradation of polyunsaturated lipids, which are the main structural substance of cellular membrane. Lipid peroxidation is a well-established mechanism of cellular injury in both plants and animals, and is used as an indicator of oxidative stress in cells and tissues. Lipid peroxides are unstable and decompose to form a complex series of compounds including reactive carbonyl compounds. Polyunsaturated fatty acid peroxides generate malondialdehyde upon decomposition [11]. Its increase in the RFA group can be explained by the significant cell destruction created by the thermal injury, corresponding to a larger extend of pulmonary tissue necrosis at both sides of the cut edge compared to the "cut and sew" method of the control group. Whether simultaneous destruction of the microcirculation in RFA lung resection could represent a restriction barrier for propagation of the regional oxidative stress to the systemic circulation needs further investigation [10].

TNF-α represents an early phase mediator of inflammation and plays a primary role in the pathogenesis of infection, tissue injury and inflammation as it is suggested by several studies in animals and humans [12-14]. It is suggested that TNF-a is synthesized by a number of cells, including monocytes/macrophages, lymphocytes, natural killer cells, glomerular mesangial cells, astrocytes and microglial cells of the brain, and Kupffer's cells of the liver. TNF-a does not exist in a stored form but is synthesized *de novo *following cell activation. The biosynthesis of TNF-α is tightly regulated by transcriptional and post-transcriptional mechanisms [17,18]. Ng et al in their research study, concerning RFA-assisted liver resection, found that during the early postoperative period, the systemic inflammatory marker concentrations (TNF-a and interleukin-1beta) in the RFA group were significantly higher than in their control group, suggesting by that, that the remnant of large necrotic radiofrequency ablated tissue can be the cause of activation of Kupffer cells. Within these cells, activation of the transcription factor complex nuclear factor B would release inflammatory factors, including TNF-α, IL-1, IL-2, IL-6, and IL-8, into the systemic circulation [19,20]. The cascade mechanism of the observed regional increase of TNF-α in our study cannot be fully explained as there is still no other equivalent study concerning evaluation of regional oxidative and pre-inflammatory stress after performing an RFA-assisted lung resection. Further study is also needed to clarify the systemic inflammatory reaction as this technique of lung resection is still under investigation.

The main limitation of our study is the small number of investigated animals together with the absence of data on the serum levels of TNF-α and MDA. This is a preliminary study as we have planned a further investigation of Total Andioxidand Status (TAS), inflammation markers and the kinetic of these substances after RFA-assisted lung resection procedures in animals. The safety limit of lung resection using the RFA technique also needs to be studied as it was proposed that RFA-assisted liver resection of more than 40% can cause significant inflammatory responses and poor survival in a rat model [10]. Furthermore, the dose-response change of systemic inflammation after RFA assisted lung resection, and the maximal host tolerance to large-volume RFA remains to be investigated.

In conclusion our data indicate that RFA-assisted wedge lung resection increases regional MDA and TNF-a factors affecting by this oxidative and pre-inflammatory stress of the procedure. Although RFA-assisted liver resection can be well tolerated in humans, the possible use of this method to the lung has to be further investigated in terms of regional and systemic reactions. The feasibility of performing larger lung resections, like lobectomy, also needs further investigation as the lung parenchyma has substantial differences from liver or kidney.

## Competing interests

The authors declare that they have no competing interests.

## Authors' contributions

ThKa, NT, NN and AP participated in the design of the study. ThKa performed the animal experimental surgery. ThKa and IA wrote the article. IA performed the statistical analysis and drafted the manuscript. ThKo participated in the statistical analysis. All authors read and approved the final manuscript.
